# Fabrication of alginate-based hydrogel cross-linked via horseradish peroxidase for articular cartilage engineering

**DOI:** 10.1186/s13104-021-05795-2

**Published:** 2021-09-28

**Authors:** Sepideh Saghati, Ali Baradar Khoshfetrat, Hamid Tayefi Nasrabadi, Leila Roshangar, Reza Rahbarghazi

**Affiliations:** 1 Department of Tissue Engineering, Faculty of Advanced Medical Sciences, Tabriz University of Medical Sciences, Tabriz, Iran; 2 Chemical Engineering Faculty, Sahand University of Technology, 51335-1996 Tabriz, Iran; 3 Stem Cell Research Center, Tabriz University of Medical Sciences, Tabriz, Iran; 4 Department of Applied Cell Sciences, Faculty of Advanced Medical Sciences, Tabriz University of Medical Sciences, 5154853431 Tabriz, Iran

**Keywords:** Alginate, Hydrogel, Phenolation, Enzymatic cross-linking

## Abstract

**Objective:**

We aimed to detect the effect of a couple of parameters including Alg, H_2_O_2_, and HRP concentrations on the gelation time of Alg-based hydrogels using an enzymatic cross-linked procedure.

**Results:**

NMR, UV–Vis, and ATR-FTIR analyses confirmed the conjugation of Ph to the Alg backbone. Data showed gelation time was delayed with the increase and reduction of H_2_O_2_ and HRP, respectively. We noted that hydrogel consisted of 1.2% (w/v) Alg, 5 U HRP, and 100 mM H_2_O_2_ yielded an appropriate gelation time with appropriate mechanical properties. The addition of 0.5% (v/v) Col developed hydrogel increased the gelation time. The data showed that Alg, HRP, and H_2_O_2_ with the ratio of 1:0.54:0.54 had proper physicochemical features for cartilage engineering.

## Introduction

In non-vascular tissues such as articular cartilage, a limited self-renewal capacity leads to delayed healing procedures. As a correlate, the development of novel therapeutic approaches for the acceleration of healing procedures in injured articular cartilage is at the center of attention [1]. Hydrogels with improved mechanical and biological properties can mechanically and biologically regulate cell bioactivity, making them suitable compounds for in vitro and in vivo conditions [2–4]. Among hydrogel polymers, natural polymers such as Alg, unbranched binary copolymers of (1–4)-linked β-d-mannuronic and α-l-guluronic residues have been used as cartilage regenerative substrates [5, 6]. Both ionic and enzymatic cross-linking procedures are suitable for the fabrication of Alg-based hydrogels by anionic gelation (CaCl_2_) and enzymatic crosslinking using phenolic groups [7, 8]. Because of ion exchanging in ionic cross-linked hydrogels, the final structures lack appropriate mechanical properties [9]. Several advantages such as mild reaction conditions (ear physiological pH and temperature) and controllable mechanical properties have doubled the importance of enzymatically cross-linked hydrogels in the fabrication of engineered scaffolds [10]. In support of this notion, Alg-based hydrogels are suitable scaffolds in the field of tissue engineering. Using different synthesis protocols and modalities, it is possible to synthesize Alg hydrogels with distinct mechanical properties comparable to the in vivo conditions [11]. Natural polymers like Col benefits from the advantages of having cell-recognizable moieties, hydrated 3D networks, lack of immunogenicity, and stimulates the cells to produce ECM [12]. Despite these superiorities, decreased mechanical stability of Col limits its extensive biological application. To bypass this obstacle, several studies have tried to combine Col with other natural components such as polysaccharides like Alg to obtain a suitable scaffold with controllable mechanical and chemical properties [13]. HRP is one of the applicable enzymes for cross-linking. HRP can react with H_2_O_2_ when added to the solution containing a polymer-phenol mixture [14]. The gelation time of the hydrogels is one of the most important factors that must be considered in controlling the final physicochemical and mechanical properties. This value depends on the reaction condition like temperature, components concentrations, cross-linking density, and the extent of Ph groups [15, 16]. Here, we aimed to synthesize hydrogel of Alg-based hydrogel with Ph groups (Alg-Ph) using the HRP-mediated cross-linking and evaluate the reaction conditions on the gelation time. The close association with hydrogel gelation time and concentrations of Alg, HRP, and H_2_O_2_ was detected. This hydrogel was designed in line with cartilage tissue engineering. Previously, we showed the chondrogenic properties of Alg-Ph-based hydrogels in the orientation of human mesenchymal stem cells toward chondrocyte-like cells in in vitro conditions [17]. Here, we tried to present improved formulation (time and concentration of components) and procedure required for an appropriate HRP-mediated cross-linked Alg-Ph hydrogel.

## Main text

### Materials and methods

#### Materials

Bovine cutaneous type I Col and HRP (P8375-1KU) were obtained from Sigma-Aldrich. The activity of HRP is based on the pyrogallol unit. Sodium Alg (molecular weight: 7  ×  10^4^; molar ratio of mannuronic acid to guluronic acid: 0.65) was purchased from KIMICA Corporation (Japan). H_2_O_2_ solution was obtained from Merck. EDC, NHS, MES, acetone, EtOH, and tyramine hydrochloride (Cat no.: T2879) were purchased from Sigma-Aldrich.

#### Synthesis of Alg-Ph

**Fig. 1:**
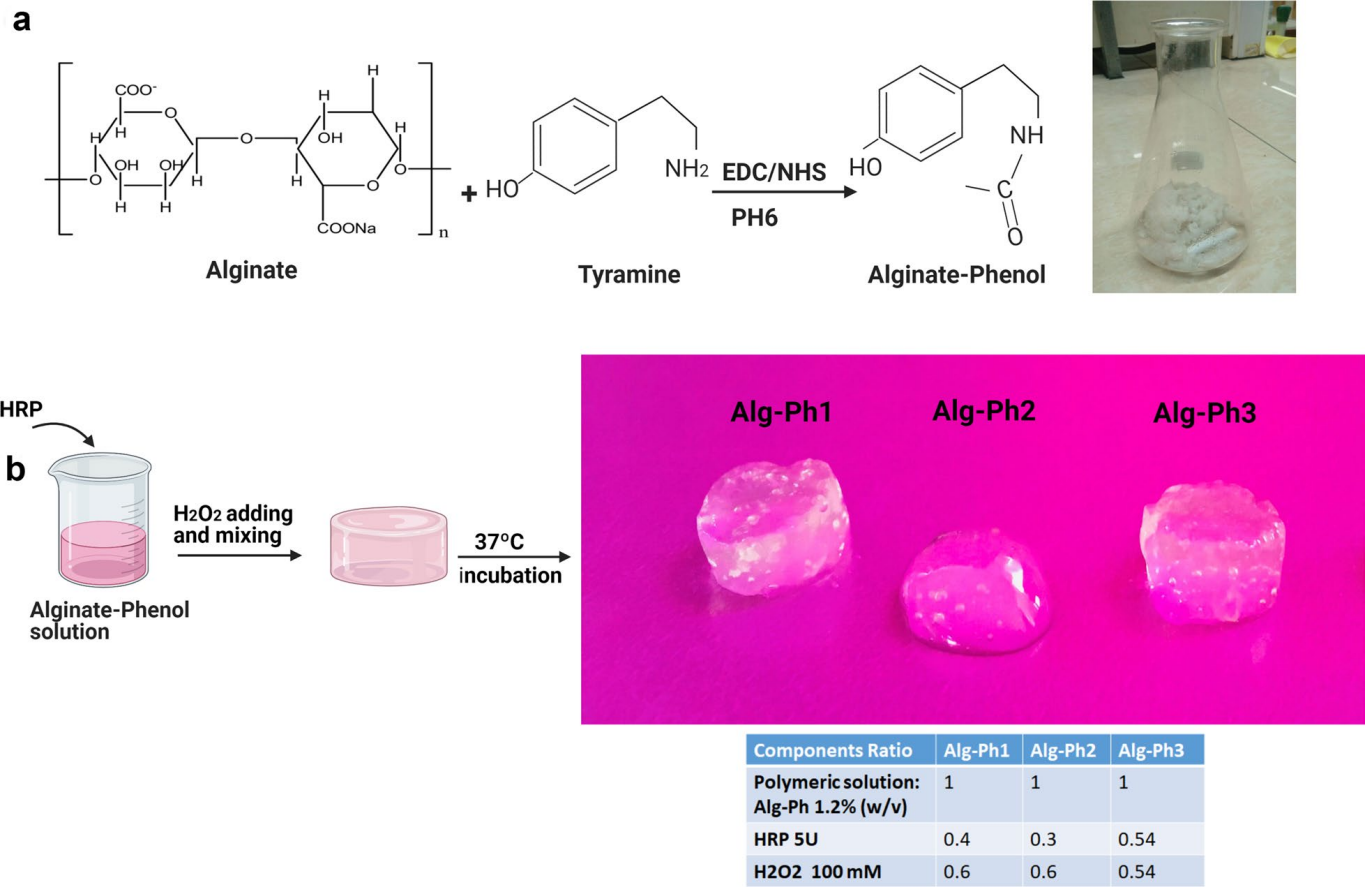
Schematic illustration of the Alg phenolation process (**a**), the Alg-Ph hydrogel fabrication (**b**)

To prepare Alg-Ph moieties, Alg was conjugated with tyramine using carbodiimide-mediated condensation [18]. To this end, we prepared 0.2% (w/v) MES and the pH value was set to 6. Alg solution (1%) was prepared by adding sodium Alg (7.5 g) to the solution. The solution was mixed until Alg completely dissolved. The process was continued by the addition of NHS (0.85 g), EDC (2.83 g), and tyramine hydrochloride (5.88 g) to the mixture. When the constituents were entirely dissolved after 20 h at 25 °C, the pH was changed to 8.6 (Fig. 1). The synthesized polymer was extracted using acetone, washed with EtOH overnight, and then lyophilized.

#### Fabrication of hydrogel

To fabricate hydrogel composed of Alg-Ph/Col, Alg-Ph powder was mixed with the 0.5% Col (w/v%) solution (Fig. 1). In this study, different HRP concentrations were used for enzymatic cross-linking of Alg-Ph. HRP was added to the Al-Ph/Col solution and mixed gently. On the other hand, different concentrations of Alg-Ph including 1, 1.2, 1.5% w/v) were used to optimize the gelation time. To synthesize a hydrogel with appropriate physical properties, different molarities of H_2_O_2_ overlaid the solution and mixed gently until complete gelation occurred. Then, the prepared hydrogel was maintained at 37 °C for 4 h. Similar to the protocol used for the synthesis of the Alg-Ph/Col hydrogel, the Alg-Ph hydrogel was also synthesized. From several synthesis protocols, those with suitable physical properties with distinct concentrations were selected for mechanical testing. These samples were named as Alg-Ph1, Alg-Ph2 and Alg-Ph3 and the final concentration of Col, Alg-Ph, HRP and H_2_O_2_ was set to 0.5% (w/v), 1.2% (w/v), 5 (units/ml), 100 (mM), respectively.

## Scaffold characterization

### ATR-FTIR

Varian 610 spectrometer (Agilent Technologies, USA) was used to detect the ATR-FTIR spectrum of Alg-Ph and Alg-Ph/Col hydrogels using a DTGS detector and a diamond crystal. Hydrogels were fabricated in cylindrical shape then frozen at − 20 °C for 24 h and lyophilized.

### Gelation time

Here, we performed gelation time assay in four different steps as follows; First, we prepared hydrogel consisted of 1.2% (w/v) Alg-Ph and 100 mM H_2_O_2_ and different concentrations of HRP, including 3, 5, 6, 8, and 10 U. Second, 1.2% (w/v) Alg-Ph and 5 U HRP were combined and different doses of H_2_O_2,_ including 15, 25, 30, 40, 50, and 100 mM were used for gelation; In the next step, different Alg-Ph concentrations (1, 1.2%, 1.5%) were added to the mixture composed of 5 U HRP plus 100 mM H_2_O_2_. After completion of these experiments, we selected hydrogels with appropriate consistency with a short gelation time. We performed the experiments with selected doses of Alg-Ph, HRP, and H_2_O_2_ and 0.5% (w/v) Col solution.

To assess gelation time, hydrogels composed of Alg-Ph and HRP were placed in culture plates (100–200 µl per 48-well plates) and gently stirred at 80 rpm. Then, H_2_O_2_ solution was added while mixing. We considered the gelation of hydrogels when the stirring was inhibited and led to the solution swelling.

### Mechanical testing

After several tryouts with various Alg, HRP, and H_2_O_2_ concentrations, we indicated that hydrogel consisted of 1.2% (w/v) Alg-Ph, 5 (u/ml) HRP, and 100 (mM) H_2_O_2_ exhibited appropriate physical stability. For mechanical testing, we used three different hydrogels as follows; Alg-Ph-1 [Alg-Ph 1.2% v/w  +  HRP 5 U  +  H_2_O_2_ 100 mM (1:0.4:0.6)], Alg-Ph-2 [Alg-Ph 1.2% v/w  +  HRP 5 U  +  H_2_O_2_ 100 mM (1:0.3:0.6)], and Alg-Ph-3 [Alg-Ph 1.2% v/w  +  HRP 5 U  +  H_2_O_2_ 100 mM (1:0.54:0.54)]. Zwick/Roell (Z010, Germany) tensile machine equipped with a 10-kN load cell was used to assess mechanical characterization of cylindrical shaped (1  ×  1 cm) hydrogels. Testing was carried out at a compression rate of 2 mm per minute. The compressive modulus of the hydrogels was calculated as the slope of a linear fit to the stress–strain curve over 5–10% strain. The test was done in triplicate.

### SEM imaging

To this end, the sample was frozen at − 80 °C for 18 h followed by lyophilization for 24 h. SEM (JEOL 840 73, Joel, Japan) was used for the assessment of morphological properties. Samples were sputter-coated and placed on a holder inside the SEM chamber.

## Results

### Gelation time

**Fig. 2:**
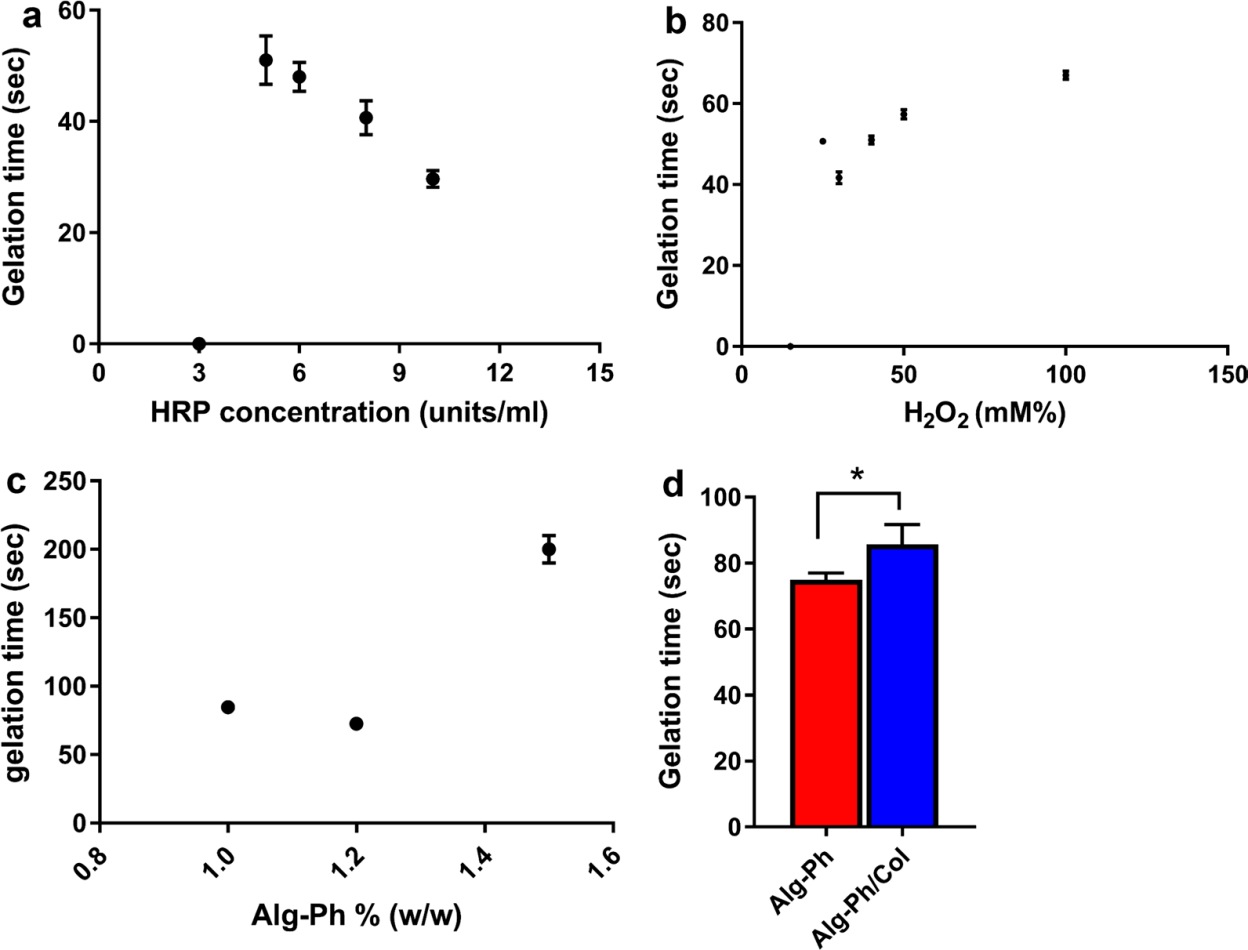
Association of gelation time for Alg-Ph hydrogels (**a**) HRP concentration (Alg-Ph 1.2% w/v, H_2_O_2_ 100 mM), (**b**) H2O2 concentration (Alg-Ph 1.2% w/v, HRP 5 U (**c**) Alg-Ph concentration, and (**d**) the combination of Alg-Ph with 0.5% (w/v) Col. Student *t* test; p  <  0.05 (n  =  3)

Here, the effect of Alg-Ph, H_2_O_2,_ and HRP concentrations was assessed on the Alg-Ph hydrogel gelation time (Fig. 2a–c). Data showed that the gelation time was reduced by decreasing the concentration of H_2_O_2_ (in the range of 3–10 sunits/ml) and increasing the HRP concentration. We noted an increased gelation time when the concentration of Alg reached 1.5% (w/v). The existence of Col in the hydrogel structure increased gelation time (Fig. 2d).

### Chemical characterization

**Fig. 3:**
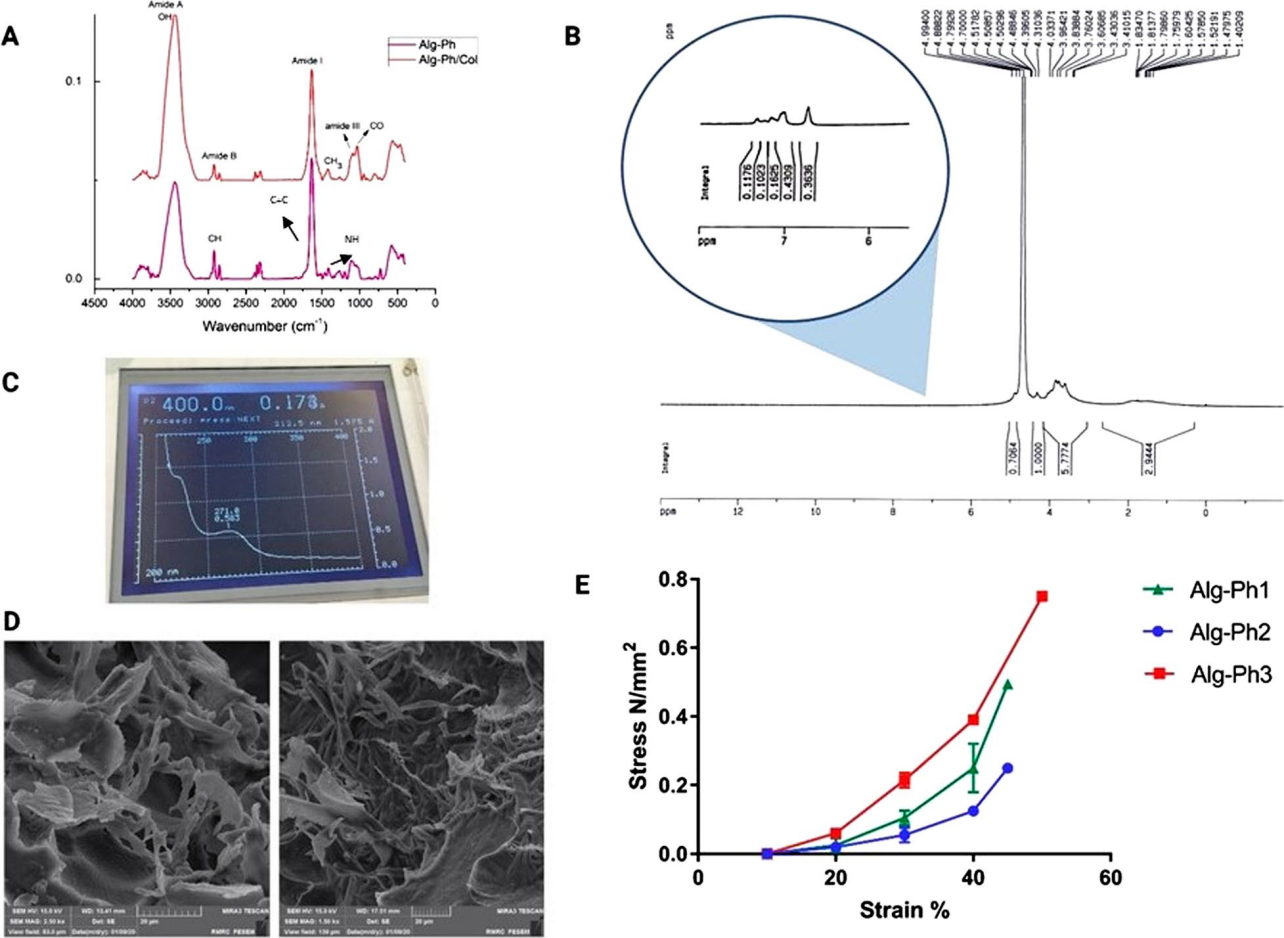
ATR-FTIR spectra of Alg-Ph and Alg-Ph/Col hydrogels (**a**). HNMR analysis of Alg-Ph powders (**b**). UV–Vis spectra of Alg-Ph (c). SEM images of Alg-Ph (left) and Alg-Ph/Col (right) (**d**). Mechanical assay for Alg-Ph-1 [Alg-Ph 1.2% v/w  +  HRP 5 U  +  H_2_O_2_ 100 mM (1:0.4:0.6)], Alg-Ph-2 [Alg-Ph 1.2% v/w  +  HRP 5 U  +  H_2_O_2_ 100 mM (1:0.3:0.6)], and Alg-Ph-3 [Alg-Ph 1.2% v/w  +  HRP 5 U  +  H_2_O_2_ 100 mM (1:0.54:0.54)] (**e**) (n  =  3)

To confirm the chemical conjugation of Ph groups with the Alg backbone, UV–Vis and NMR analyses were performed. The chemical composition of Alg-Ph and Alg-Ph/Col hydrogels was determined using ATR-FTIR analysis (Fig. 3a). The FTIR spectra indicate the characteristic peaks of Alg and Col including strong band at 1630–1634 cm^−1^ proving the C = C groups of tyramine, hydroxyl O–H broadband in the range of 3000–3600 cm^−1^, ether aliphatic C–H stretching band (2900–2850 cm^−1^), and stretching vibrations of carboxylic functional groups related to aliphatic ether (1190 cm^−1^) [17]. Also, the spectra of Alg-Ph/Col exhibited the Col fingerprint bands at 1260–1270 cm^−1^ related to the C–N and N–H bands, 1630 cm^−1^ (typical of amide I), 3418 cm^−1^ (amide A), and 2930 cm^−1^ (amide B). According to the NMR analysis, distinguishing peak areas of Ph were observed at 6.6–7.2 ppm (Fig. 3b) [19]. The peaks related to protons on carbons adjacent to a carboxylic acid were detected in the 2–3 ppm region. The spectra represent characteristic peaks of Alg polymer at 4–5 ppm. The UV–Vis spectra of the Alg-Ph solution were detected in an aqueous solution at 275 nm which is in agreement with the NMR results and associated with Ph groups (Fig. 3c) [20].

### Morphological investigations

SEM imaging showed homogeneous microstructure and improved interconnectivity of Alg-Ph and Alg-Ph/Col hydrogels (Fig. 3d). As shown in Fig. 3d, Alg and Col micro-fibrils are recognizable throughout the structure. These data confirm the capability of our methodology to fabricate hydrogels with appropriate pore size and homogenous porosity for cell seeding applications.

### Mechanical characterization

Figure 3e illustrates the compressive mechanical properties of three final selected compositions named Alg-Ph1, Alg-Ph2, and Alg-Ph3. The data showed that the Alg-Ph3 hydrogel had the highest compressive strength among three samples with the ultimate compressive strength of 750 (KPa) and broke at 49–50% strain. While the lowest stability was related to the Alg-Ph2 hydrogel broke at 45% strain with an ultimate compressive strength of 260 (KPa). According to the data, the highest compressive modulus was exhibited by the Alg-Ph3 hydrogel (13.4 MPa compared with 11.5 MPa for Alg-Ph1 and Alg-Ph2).

## Discussion

Alg-Ph was synthesized via the peroxidase-catalyzed oxidative crosslinking method. The relation between hydrogels gelation time and concentration of components was assessed. We performed different combinations and formulas with various concentrations of Alg, HRP, and H_2_O_2_ to find appropriate gelation time. Gelation time is an incredible factor for hydrogel evaluation [21]. Data showed that the longer gelation times result from the enhanced concentration of H_2_O_2_ and decreased concentration of HRP which can be explained as a consequence of the deactivation of HRP by H_2_O_2_. To select a composition based on appropriate gelation time (as an optimizing factor), several items including mechanical and morphological properties should be considered. On the other hand, it should be noted that H_2_O_2_ is a strong oxidant with a damaging effect; a decreased gelation time with a lower concentration of H_2_O_2_ is an appropriate chemical agent for biological applications. It should be considered that in the HRP-mediated cross-linking method, Ph derivatives are used because of electron donor capacity. In this reaction, HRP reacts with H_2_O_2_ quickly, and the consequential compound can oxidize phenolic hydroxyl (Ph) groups [16]. Therefore, another important factor that influences the gelation time is the level of Ph groups in Alg-based hydrogel. We examined three different concentrations of Alg-Ph 1, 1.2, and 1.5% (v/w) and monitored gelation time. Figure 3 shows that the lowest value for gelation time is achievable at the concentration of 1.2%. The elevation of Alg-Ph concentration led to delayed gelation time. This effect is associated with the existence of excessive amounts of unreacted Ph groups and can produce soft gels with extremely low mechanical stability. We found that the addition of Col to the Alg-Ph hydrogel increases the gelation time. It is suggested that the distribution of Col microfibrils inside the hydrogel supports platform to react with Alg-Ph molecules thus limits the availability of Ph groups to HRP/H_2_O_2_. After several technical replicates, we found that hydrogel composed of 1.2% (w/v) Alg-Ph, 5 (u/ml) HRP, and 100 (mM) H_2_O_2_ exhibited appropriate stability for fabrication of cartilage-like engineered scaffold. To find the final concentration, three compositions were selected and investigated by mechanical testing. The results showed that the Alg-Ph3 hydrogel had the highest ultimate strength, compressive modulus, and strain in comparison with Alg-Ph 1 and 2 groups. In the optimized values, HRP and H_2_O_2_ efficiently interact with each other in the presence of Ph groups.

## Conclusion

Alg is a versatile polymer that can be used for an extensive range of biomedical applications. Enzymatic cross-linking of Alg improves the flexibility of the final hydrogel and yielded favorable mechanical properties. We found that mechanical and physical properties of 1.2% (v/w) Alg-Ph, 5 U HRP, 100 mM H_2_O_2_ at the ratio of 1:0.54; 0.54 are appropriate for cartilage-like engineered scaffolds.

## Limitation

This work faces numerous limitations. Further investigations are needed to assess different concentrations of hydrogel H_2_O_2_, HRP, and Alg-Ph. It is suggested to monitor the gelation time and mechanical properties of Alg-based hydrogels after the addition of other ECM components. Besides, we did not examine the developed hydrogel in in vivo conditions. It should not be forgotten that the flexibility and consistency of Alg-Ph-based hydrogel can be different inside the body compared to the in vitro conditions.

## Data Availability

The data created and analyzed during the current study are available from the corresponding author upon reasonable requests.

## Abbreviations

EDC: 1-Ethyl-3-(3-dimethylaminopropyl) carbodiimide
MES: 2-Morpholinoethanesulfonic acid, monohydrate
Alg: Alginate
Col: Collagen
EtOH: Ethanol
ATR-FTIR: Fourier transform infrared spectroscopy
HRP: Horseradish peroxidase
H_2_O_2_: Hydrogen peroxide
NHS: N-hydroxysuccinimide
NMR: Nuclear magnetic resonance
Ph: Phenol
SEM: Scanning electron microscopy
UV–Vis: Ultraviolet–visible spectroscopy
